# LncRNA H19 ameliorates myocardial infarction‐induced myocardial injury and maladaptive cardiac remodelling by regulating KDM3A

**DOI:** 10.1111/jcmm.14846

**Published:** 2019-11-21

**Authors:** Bo‐fang Zhang, Hong Jiang, Jing Chen, Qi Hu, Shuo Yang, Xiao‐pei Liu, Gen Liu

**Affiliations:** ^1^ Department of Cardiology Renmin Hospital of Wuhan University Wuhan China; ^2^ Cardiovascular Research Institute Wuhan University Wuhan China; ^3^ Hubei Key Laboratory of Cardiology Wuhan China

**Keywords:** KDM3A, LncRNA H19, miR‐22‐3p, myocardial infarction

## Abstract

Myocardial infarction (MI) remains the leading cause of morbidity and mortality worldwide, and novel therapeutic targets still need to be investigated to alleviate myocardial injury and the ensuing maladaptive cardiac remodelling. Accumulating studies have indicated that lncRNA H19 might exert a crucial regulatory effect on cardiovascular disease. In this study, we aimed to explore the biological function and molecular mechanism of H19 in MI. To investigate the biological functions of H19, miRNA‐22‐3p and KDM3A, gain‐ and loss‐of‐function experiments were performed. In addition, bioinformatics analysis, dual‐luciferase reporter assays, RNA immunoprecipitation (RIP) assays, RNA pull‐down assays, quantitative RT‐PCR and Western blot analyses as well as rescue experiments were conducted to reveal an underlying competitive endogenous RNA (ceRNA) mechanism. We found that H19 was significantly down‐regulated after MI. Functionally, enforced H19 expression dramatically reduced infarct size, improved cardiac performance and alleviated cardiac fibrosis by mitigating myocardial apoptosis and decreasing inflammation. However, H19 knockdown resulted in the opposite effects. Bioinformatics analysis and dual‐luciferase assays revealed that, mechanistically, miR‐22‐3p was a direct target of H19, which was also confirmed by RIP and RNA pull‐down assays in primary cardiomyocytes. In addition, bioinformatics analysis and dual‐luciferase reporter assays also demonstrated that miRNA‐22‐3p directly targeted the KDM3A gene. Moreover, subsequent rescue experiments further verified that H19 regulated the expression of KDM3A to ameliorate MI‐induced myocardial injury in a miR‐22‐3p‐dependent manner. The present study revealed the critical role of the lncRNAH19/miR‐22‐3p/KDM3A pathway in MI. These findings suggest that H19 may act as a potential biomarker and therapeutic target for MI.

## 1 INTRODUCTION

Coronary heart disease is characterized by the formation of stable atheromas that induce chronic myocardial ischaemia or of vulnerable plaques that lead to acute occlusive atherothrombotic complications.^1^ Acute myocardial infarction (AMI), commonly known as heart attract, often occurs as the most serious and lethal manifestation of coronary heart disease.^2^ The heart is extremely sensitive to ischaemic or hypoxic injury, and a rapid and persistent decrease in coronary artery flow secondary to coronary artery occlusion results in irreversible cardiomyocyte loss, leading to subsequent heart failure and even sudden cardiac death.^3^ Despite significant progress in the treatment and management of the disease as well as improved secondary prevention measures, AMI remains the leading cause of disability and mortality worldwide.^2^ Currently, the most effective strategy for the treatment of AMI mainly focuses on timely reperfusion of the ischaemic myocardium by dissolving the thrombus with medications or percutaneous coronary intervention.^4^ Although these strategies are effective in limiting heart injury and reducing infarct size, patients with AMI remain at increased short‐term and long‐term risk of heart failure, and only approximately half of patients survive for 5 years or longer after diagnosis.^5^ Therefore, therapeutic approaches that could alleviate progressive maladaptive cardiac remodelling and improve heart performance in the long run are still the Holy Grail of this field.

The discovery of non‐coding RNAs has provided a new perspective on gene regulation in multiple pathophysiological contexts. Among them, the biological functions of microRNAs have been extensively investigated in cardiovascular disease over the last decade. However, much less is known about the functional role of lncRNAs in this field.^6^ LncRNAs are currently defined as transcripts that are longer than 200 nucleotides without evident protein‐coding function. Thus far, lncRNAs have been perceived as involved in myriad biological processes and exert powerful regulating efficiency at the epigenetic, transcriptional and post‐transcriptional levels.^7^ As the homeostasis of the cardiovascular system requires the exquisite control of gene expression and transcriptional programs, it is reasonable to speculate that lncRNAs might emerge as crucial mediators in cardiovascular diseases.

LncRNA H19 is located near the telomeric region of human chromosome 11 in the H19 gene, which is one of the most well‐known imprinted genes.^8^ H19 is abundantly expressed in embryonic tissues of endodermal and mesodermal origin, and it has been demonstrated to be expressed mainly in skeletal muscle and the heart in adults.^9^ H19 has been deemed to be involved in heart development and blood vessel formation.^10^ In addition, several prospective experiments have also indicated that H19 plays a vital role in cardiovascular diseases, such as myocardial ischaemia‐reperfusion injury, cardiac hypertrophy and aortic calcification.^11^, ^12^, ^13^ Moreover, aberrant expression of H19 has been identified in AMI patients in clinical studies.^14^ However, whether H19 participates in the pathological progress of AMI and its potential function remain largely obscure.

Lysine (K)‐specific demethylase 3A (KDM3A), also named Jumonji domain‐containing 1A (JMID1A), is an important epigenetic mark that exerts its biological effects by specifically demethylating mono‐methyl and di‐methyl histone H3K9 (H3K9me1/2) and consequently modulating target gene expression.^15^ As H3K9 methylation in promoter regions is usually regarded as a suppressive histone mark that inhibits transcription, the role of demethylase KDM3A tends to be as an epigenetic activator that facilitates gene transcription. Numerous studies have elucidated the pathophysiological significance of KDM3A in various tumours as a poor prognostic indicator^16^; however, its potential function in cardiovascular disease is still enigmatic. Our previous studies have provided firm evidence that KDM3A is implicated in diabetic vascular remodelling and diabetic cardiomyopathy as well as myocardial ischaemia‐reperfusion injury by regulating key signalling pathways that are involved in inflammation, apoptosis and oxidative stress.^15^, ^17^ In addition, Zhang and colleagues also reported that KDM3A participated in left ventricular hypertrophy and myocardial fibrosis in response to pressure overload.^18^ In the light of these considerations, we speculated that KDM3A may play a central role in the regulatory network of AMI.

The present study was designed to determine whether lncRNA H19 was involved in the pathophysiological process of AMI as well as to explore the underlying molecular biological mechanisms. Using an in vivo rat AMI model, our experiment provides compelling evidence that H19 could modulate the expression of KDM3A by competitively binding to miR‐22‐3p, which consequently ameliorated AMI‐induced myocardial damage and cardiac remoulding.

## 2 MATERIALS AND METHODS

### 2.1 Reagents

Dulbecco's modified Eagle's medium (DMEM/F12), foetal bovine serum (FBS) and collagenase II were obtained from HyClone. Trypsin was provided by Beyotime. All other cell culture reagents were purchased from Sigma Chemicals unless otherwise specified. Primary antibodies against KDM3A, ETS‐1, Bax, Bcl‐2, IL‐6, TNF‐α, GAPDH, ANP and BNP were purchased from Abcam. The secondary antibodies, either HRP‐linked goat anti‐mouse IgG or goat anti‐rabbit IgG, were also obtained from Abcam. The miR‐22‐3p mimic and its scrambled oligonucleotides (miRNA‐NC) were synthesized by GenePharma Co., Ltd.

### 2.2 Animals

Male Sprague Dawley (SD) wild‐type rats (SPF grade, 200‐250 g) and neonatal rats (1‐3 day) were obtained from Wuhan University Experiment Animal Center. All experimental procedures were approved by the Animal Care and Use Committee of Wuhan University and performed in accordance with the Guide for the Care and Use of Laboratory Animals published by the US National Institutes of Health (NIH Publication, 8th Edition, 2011).

### 2.3 KDM3A knockout rats

The KDM3A gene knockout rat (SD background) was conducted by using CRISPR/Cas9 genome‐editing technology. In brief, one single‐guide RNA (sgRNA) flanking exon 5 of the KDM3A gene in rats was designed. Then, purified Cas9 mRNA and the sgRNA were mixed and microinjected into embryos. PCR products were TA cloned and sequenced to define the exact insertion/deletion mutations of the generated founder rats. The founder rats with multiple mutated alleles were identified and mated with the wild‐type SD rat (KDM3A+/+) strain to generate F1 heterozygotes (KDM3A+/−). Thereafter, KDM3A gene deletion homozygote rats (KDM3A−/−) were obtained through sibling mating of the heterozygous F1 generation. The KDM3A‐KO rats were identified and screened by sequencing of PCR products. Western blot assays were performed to verify KDM3A protein expression as well.

### 2.4 Adenoviral constructions and transfection

The following were inserted into adenoviral vectors according to the manufacturer's instructions (GeneChem): the full‐length rat lncRNA H19 sequence (Ad‐H19) to ensure H19 expression, a short‐hairpin RNA (Ad‐shH19) directed against lncRNA H19 and the associated negative control oligonucleotides. Recombinant adenoviruses for miR‐22‐3p overexpression (Ad‐miR‐22) and miR‐22‐3p silencing (Ad‐Antagomir‐22), as well as their negative control, were also constructed by GeneChem. The adenovirus encoding KDM3A (Ad‐KDM3A), which has been verified to be efficient in inducing the overexpression of KDM3A in vivo,^15^ was provided by GeneChem as well, and Ad‐GFP was used as a control.

Five days before the establishment of the AMI model, rats were anaesthetized, and the pericardium was removed through a small left anterior thoracotomy. Then, 100 µL of adenovirus solution was intramyocardially injected into five separate sites of the left ventricle by using a microsyringe. Five days later, the rats that survived were subjected to AMI surgery.

### 2.5 Establishment of the rat AMI model

The rat AMI model was established as previously described.^19^ Briefly, animals were anaesthetized with intraperitoneally injected pentobarbital sodium (60 mg/kg). Then, a volume‐controlled small animal respirator was used to support the breathing of the rats through a tracheal cannula, and a standard electrocardiogram was used to continuously monitor the heart rate. Subsequently, a left thoracotomy was performed to expose the heart, and a 6‐0 silk suture on a small curved needle was passed through the myocardium beneath the left arterial descending (LAD) branch of the coronary artery. The LAD was ligatured to induce myocardial ischaemia. A successful AMI model was confirmed by an elevated ST segment in leads‐II and a regional paleness of the myocardial surface. Rats in the sham‐operated group were subjected to the same procedure except the snare was left untied. Four weeks after surgery, the rats were killed, and the hearts were harvested for further analysis.

### 2.6 Isolation and culture of primary cardiomyocytes

Primary neonatal cardiomyocytes were isolated from the heart of 1‐3‐day‐old rats and cultured in DMEM/F12 containing 15% FBS. In brief, hearts were rapidly removed from anaesthetized infant rats, and the ventricular myocardium was dissected into small pieces under sterile conditions. After digestion with 0.125% trypsin for 10 minutes, the myocardial tissue was collected and continued to be digested with a mixed enzyme solution containing 0.125% trypsin and 0.08% collagenase II at 37°C until there was no obvious tissue left. The digested cells in the supernatant were collected and centrifuged at 1000 rpm for 10 minutes and then pre‐plated into cell culture dishes for 90 minutes to separate cardiomyocytes from cardiac fibroblasts by adherence. At the end of the differential adhesion process, the non‐attached cardiomyocytes that were suspended in DMEM were collected and reseeded into cell culture dishes with medium containing 15% FBS and 1% penicillin/streptomycin for 72 hours before any subsequent treatment.

### 2.7 Assessment of heart morphology and cardiac function

Four weeks after MI surgery, rat hearts of each group were extirpated and transected at the plane of the papillary muscle, followed by freezing in an OCT compound. Sections of 10 μm thickness were obtained every 100 μm, and Masson's trichrome staining was performed according to the manufacturer's instructions. Thereafter, images were acquired using a Pannoramic MIDI Slide Scanner. The percentage of infarcted areas in each section was calculated.

Transthoracic echocardiography was performed on rats lightly anaesthetized by inhaling isoflurane. Left ventricular function at the end of the experiment was measured by using the MyLab 30CV ultrasound system (BiosoundEasote, Inc). Two‐dimensional guided M‐mode tracings were recorded in short‐axis views at the level of mid‐papillary muscles. Then, the left ventricular ejection fraction (LVEF) and left ventricular fractional shortening (LVFS) were measured. All measurements were made from at least three representative cycles and averaged.

### 2.8 Evaluation of myocardial apoptosis and fibrosis

After the animals were killed, myocardial samples of each group were fixed in 4% paraformaldehyde and embedded in paraffin. The apoptosis of cardiomyocytes was measured by TUNEL staining according to the manufacturer's instructions (Roche Applied Science). Anti‐α‐actinin antibody was used to identify the cardiomyocytes, while red fluorescent dye and DAPI were used to label the apoptotic nuclei and normal nuclei, respectively. At least five randomly selected fields per section were observed under a fluorescence microscope (400× magnification). The apoptosis index (AI) was calculated as the ratio of TUNEL‐positive cells (red) to total cardiomyocytes (blue). Myocardial remodelling after infarction was determined by Masson's staining as previously described.^19^ Areas stained in blue indicate the deposition of collagen fibrils. All histological assessments were performed by an investigator who was blinded to the experiment.

### 2.9 Luciferase reporter assay

Bioinformatics analysis predicted that H19 contains canonical binding sites for miR‐22‐3p. Meanwhile, bioinformatics analysis also indicated that KDM3A is a potential target gene of miR‐22‐3p. To verify whether they could directly interact with each other, we constructed the recombinant plasmids pmirGLO‐H19‐WT/pmirGLO‐H19‐Mut and pmirGLO‐KDM3A‐WT/pmirGLO‐KDM3A‐Mut. The rat H19 wild‐type sequence (H19‐WT) and the mutant derivative lacking the miR‐22‐3p binding site (H19‐Mut) were subcloned downstream of the coding region of the luciferase gene. The wild‐type (WT) KDM3A 3’‐UTR fragment containing putative miR‐22‐3p binding sites and its mutated form were also cloned immediately downstream of the coding region of the luciferase gene. HEK293T cells were seeded into 24‐well plates and cotransfected with the luciferase reporter plasmids and miR‐22‐3p mimic or miR‐NC using Lipofectamine 2000 (Invitrogen) according to the manufacturer's instructions. 48 hours after transfection, a dual‐luciferase reporter assay system (Promega) was used to examine the luciferase activity in each group.

### 2.10 RNA immunoprecipitation (RIP) assay

A RIP assay was performed using a Magna RIP™ RNA‐Binding Protein Immunoprecipitation Kit (Millipore) following the manufacturer's instructions as described in a previous study.^20^ Cardiomyocytes were lysed in RNA lysis buffer containing protease and RNase inhibitors. Then, a portion of the cell extract was isolated as the input, while the other portion was incubated with an antibody for coprecipitation by incubation with RIP immunoprecipitation buffer containing magnetic beads conjugated with anti‐Argonaute protein 2 (Ago‐2) antibody (Millipore) or control anti‐IgG antibody (Millipore) as a negative control. The samples were then incubated with proteinase K to digest the unbound proteins, and immunoprecipitated RNA was isolated. Subsequently, qRT‐PCR assays were conducted to determine H19 and miR‐22‐3p levels in the precipitates.

### 2.11 Pull‐down assay with biotinylated miR‐22‐3p

Cardiomyocytes were transfected with biotinylated miR‐22‐3p‐WT or biotinylated miR‐22‐3p‐Mut. After incubation for 48 hours, cells were collected and lysed with lysis buffer. Cells lysates were then incubated with streptavidin‐coated magnetic beads (M‐280 Dynabeads; Invitrogen). To prevent non‐specific binding of RNA and protein complexes, the beads were also coated with RNase‐free bovine serum albumin (BSA) and yeast tRNA (Sigma). After incubation at 4°C for 3 hours, the beads were rinsed with ice‐cold lysis buffer and washed three times with low‐salt buffer and once with high‐salt buffer. The bound RNAs were purified using TRIzol, and the level of H19 was analysed.

### 2.12 Pull‐down assay with biotinylated H19

To identify whether H19 could pull down miR‐22‐3p, the cardiomyocytes were also transfected with biotin‐labelled Bio‐probe NC, Bio‐LncRNA H19‐WT and Bio‐LncRNA H19‐Mut. 48 hours later, the cells were lysed and incubation with M‐280 streptavidin magnetic beads as described above. Afterwards, the beads were washed with pre‐cooled lysis buffer, low‐salt buffer and high‐salt buffer in sequence. Subsequently, the RNA complexes bound to the beads were purified by TRIzol, the level of miR‐22‐3p was tested by RT‐PCR.

### 2.13 Quantitative RT‐PCR and Western blot analyses

Quantitative RT‐PCR (qRT‐PCR) was performed as previously described.^19^ The primer sequences for the real‐time RT‐PCR are listed as follows: collage I forward: 5′‐TGACTGGAAGAGCGGAGAGT‐3′, collage I reverse: 5′‐GAATCCATCGGTCATGCTCT‐3′; collage III forward: 5′‐TTTGTGCAATGTGGGACCTG‐3′, collage III reverse: 5′‐AATGGGATCTCTGGGTTGGG‐3′; TGF‐β forward: 5′‐CACTCCCGTGGCTTCTAGTG‐3′, TGF‐β reverse: 5′‐GGACTGGCGAGCCTTAGTTT‐3′; GAPDH forward: 5′‐ACAGCAACAGGGTGGTGGAC‐3′; GAPDH reverse: 5′‐TTTGAGGGTGCAGCGAACTT‐3′; miR‐22‐3p forward: TGCTGCCAGTTGAAGAACTGT; reverse: CTCAACTGGTGTCGTGGAGTC; U6 forward: CCTGCTTCGGCAGCACAT, U6 reverse: AACGCTTCACGAATTTGCGT. H19 forward, 5′‐TAAAGCAGCTGGGGTGGTGAG‐3′; H19 reverse: 5′‐TGACTGGCAGGCACATCCAC‐3′. GAPDH served as the internal control for mRNA and lncRNA expression, while U6 was used as endogenous controls for miRNA expression.

For the Western blot assay, myocardial samples were lysed and proteins were extracted according to the specification of manufacturers. Total proteins were separated by 10% SDS‐PAGE and electrophoretically transferred onto polyvinylidene fluoride (PVDF) membranes (Millipore). Then, 5% non‐fat dried milk was used to block non‐specific binding to the membranes, and the membranes were incubated with primary antibodies overnight, followed by incubation with the corresponding secondary antibodies. The protein bands were visualized using an enhanced chemiluminescence system (Thermos Fisher Scientific, Inc). GAPDH was used as an internal loading control.

### 2.14 Statistical analysis

Continuous values were presented as mean ± standard deviation (SD). Student's *t‐*test was used for comparisons between two groups. One‐way analysis of variance (ANOVA) was used for comparisons among multiple groups, and Student‐Newman‐Keuls (SNK)‐q tests were used for subsequent analysis between multiple comparisons. *P*‐value of <.05 was considered statistically significant. All statistical analyses were performed using SPSS 19.0 software.

## 3 RESULTS

### 3.1 LncRNA H19 overexpression attenuated while lncRNA H19 inhibition exacerbated myocardial injury caused by MI

**Figure 1:**
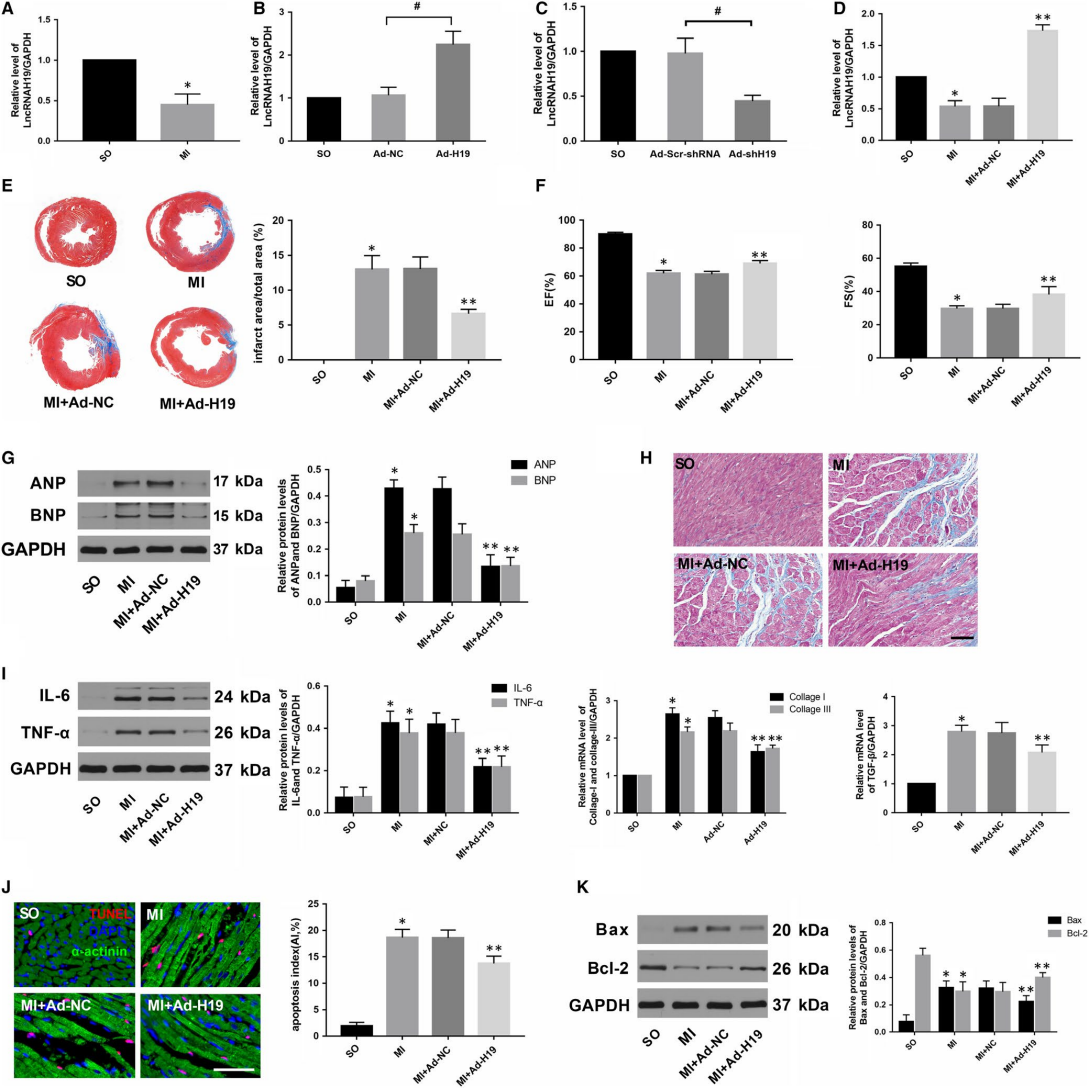
Enforced H19 expression prominently alleviated AMI‐induced myocardial injury and ensuing maladaptive cardiac remodelling. A, qRT‐PCR analysis of H19 levels in normal myocardial tissue or in the infarction border zone. (B, C, D) Transfection efficiencies of adenoviruses were determined by qRT‐PCR, ^#^ *P* < .05. E, Representative Masson's staining images at the heart papillary muscle cross‐sectional scan and the infarct rate of the different groups. F, LVEF and LVFS 4 wk after AMI in the different groups. G, Western blot analysis of the expression of ANP and BNP 4 wk after AMI in the different groups. H, Representative images of Masson's staining (scale bar = 50 μm) and mRNA expression of collagen I, collagen III and TGF‐β in the different groups. I, Western blot analysis of the expression of IL‐6 and TNF‐α in the different groups. J, Representative images of TUNEL staining in the different groups (scale bar = 50 μm): DAPI‐labelled nuclei of cardiomyocytes (blue); ɑ‐actinin‐labelled cardiomyocytes (green); TUNEL‐labelled nuclei of apoptotic cardiomyocytes (red). K, Western blot analysis of the expression of Bax and Bcl‐2 in the different groups is shown. The values were expressed as the mean ± standard deviation (SD). *P < .05 versus the SO group; **P < .05 versus the MI + Ad‐NC group

To determine whether H19 is involved in the pathological process of MI, we first assessed H19 levels in myocardial tissue 4 weeks after MI. As shown in Figure 1A, H19 expression was much lower in the myocardium of the infarcted border zone when compared with that in the normal left ventricle. To further explore the impact of H19 on MI, we performed controlled gain‐ and loss‐of‐function experiments to identify the effects of H19 in vivo. Five days before the MI surgery, rats were intramyocardially injected with either Ad‐H19 or Ad‐shH19 to stably up‐regulate or down‐regulate H19 levels, respectively (Figure 1B,C). At the end of the experiments, morphology and cardiac function as well as related molecular biological alterations in each group were rigorously scrutinized.

**Figure 2:**
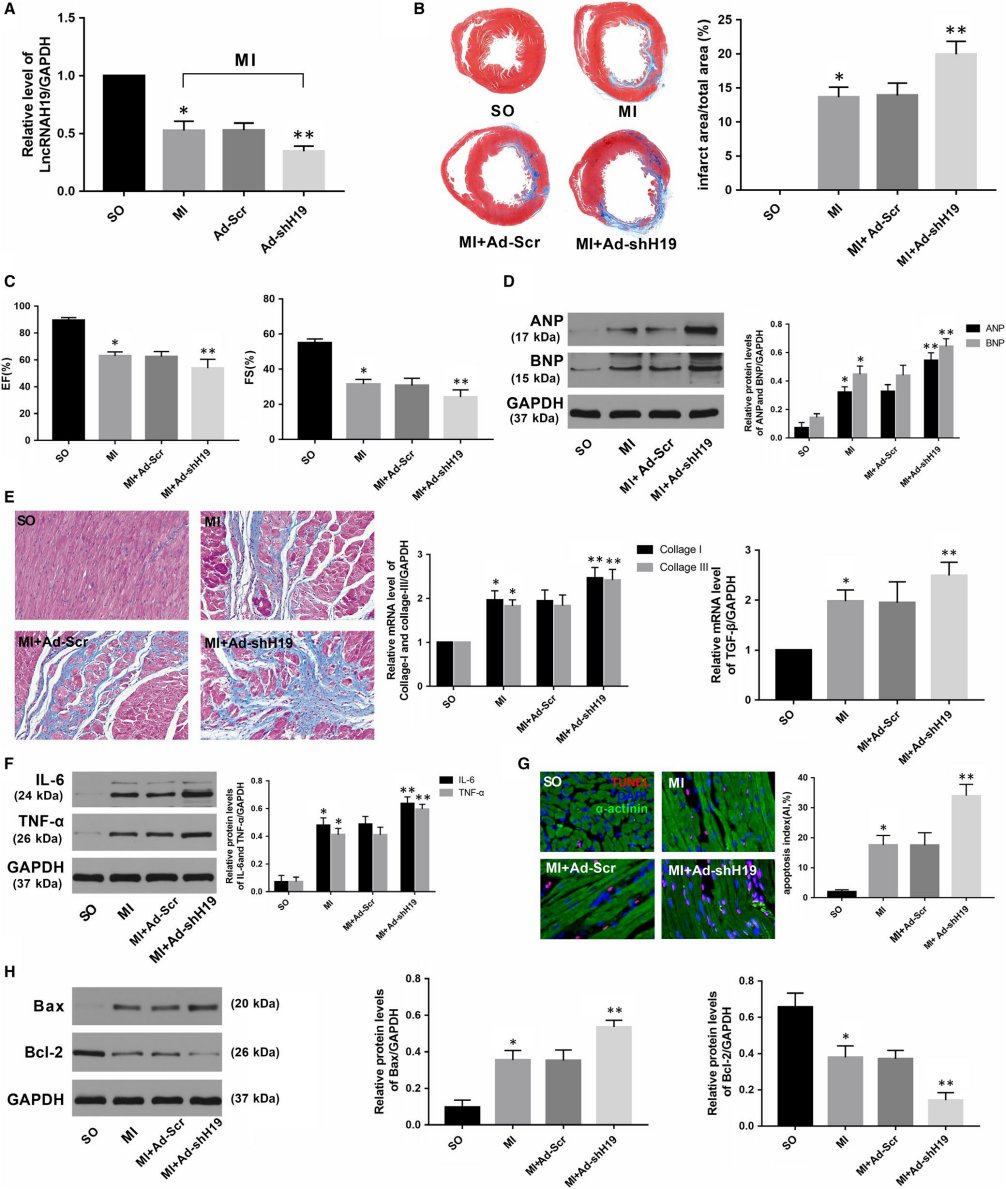
H19 knockdown exacerbated AMI‐induced myocardial injury and the ensuing maladaptive cardiac remodelling. A, The transfection efficiency of Ad‐shH19 was determined by qRT‐PCR. B, Representative Masson's staining images of the heart papillary muscle cross‐sectional scan images and the infarct rate of the different groups. C, EF and FS 4 wk after AMI. D, The expression of ANP and BNP 4 wk after AMI. E, Representative images of Masson staining (scale bar = 50 μm) and the mRNA expression of collagen I, collagen III and TGF‐β in the different groups. F, The expression of IL‐6 and TNF‐α. G, Representative images of TUNEL staining and AI in the different groups (scale bar = 50 μm). H, The expression of apoptosis‐related protein in the different groups. The values are expressed as the mean ± SD. *P < .05 versus the SO group; **P < .05 versus the MI + Ad‐Scr group

As shown in Figure 1D, transfection with Ad‐H19 significantly increased H19 levels after MI (*P* < .05). In addition, Masson's trichrome staining at the papillary muscle plane suggested an obvious reduction in infract size in Ad‐H19‐infected MI rats (Figure 1E; *P* < .05). Echocardiography results also indicated that H19 overexpression notably improved cardiac function as evidenced by increased LVEF and LVFS compared with those in the MI + Ad‐NC group (Figure 1F, *P* < .05). Moreover, the protein levels of ANP and BNP, which served as indicators of cardiac function, were also markedly decreased in rats transfected with Ad‐H19 (Figure 1G, *P* < .05). In addition, data also indicated that up‐regulated H19 could alleviate myocardial fibrosis as well as decrease the expression of pro‐fibrogenic mediators such as collagen I, collagen III and TGF‐β (Figure 1H, *P* < .05). Inflammation and apoptosis are commonly accepted as the underlying mechanisms that contribute to MI. As shown in Figure 1I, the expression of classic inflammatory cytokines such as IL‐6 and TNF‐α was dramatically decreased in the Ad‐H19 + MI group compared with the Ad‐NC + MI group (*P* < .05). Similarly, much less TUNEL‐positive cells were observed in the myocardium of the Ad‐H19 + MI group compared with the Ad‐NC + MI group (Figure 1J, *P* < .05). Moreover, H19 overexpression also prominently down‐regulated the level of the pro‐apoptosis protein Bax while it up‐regulated the level of the anti‐apoptotic protein Bcl‐2 (Figure 1K; *P* < .05). However, the outcome was distinctly reversed when the heart was infected with Ad‐shH19 to knock down H19 expression. As shown in Figure 2B,C,D and E, H19 inhibition further enlarged the infract size, deteriorated the compromised cardiac function and aggravated the degree of myocardial fibrosis (*P* < .05). Moreover, H19 knockout dramatically exacerbated inflammation and myocardial apoptosis, as evidenced by enhanced levels of inflammation and apoptosis‐associated parameters (Figure 2F,G and H; *P* < .05). Therefore, the above gain‐ and loss‐of‐function experiments strongly suggest that H19 may act as a therapeutic target of MI.

### 3.2 LncRNA H19 could directly interact with miR‐22‐3p and regulate its expression

**Figure 3:**
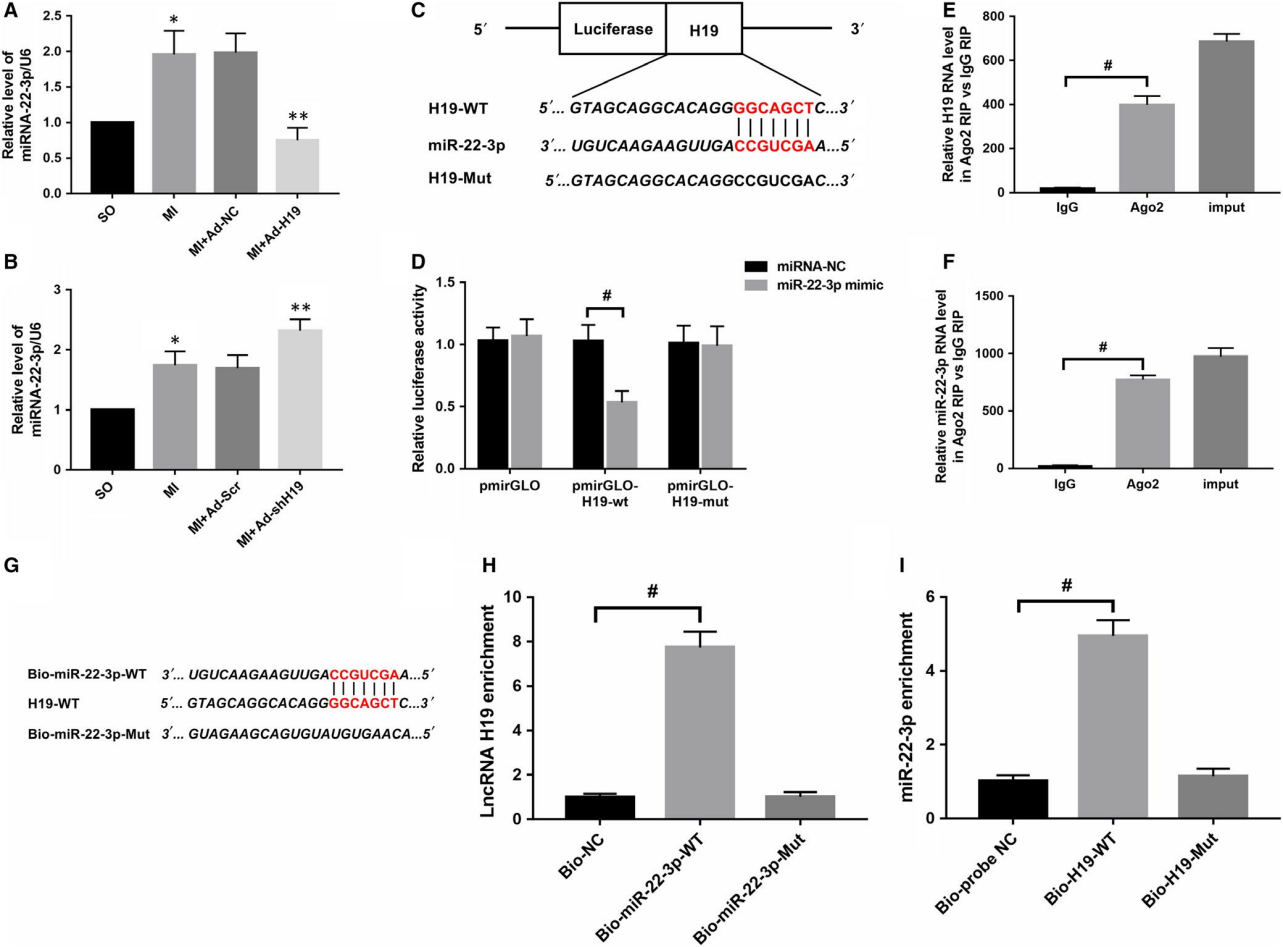
miRNA‐22‐3p is a direct target of H19 in cardiomyocytes. A, The miRNA‐22‐3p levels were measured by qRT‐PCR after up‐regulation of H19 expression by adenovirus infection. *P < .05 versus the SO group; **P < .05 versus the MI + Ad‐NC group. B, Levels of miRNA‐22‐3p after down‐regulation of H19 expression by adenovirus infection. *P < .05 versus the SO group; **P < .05 versus the MI + Ad‐Scr group. C, H19 contains a site complementary to miRNA‐22‐3p. D, Luciferase activity in HEK293T cells cotransfected with pmirGLO‐H19‐Wt or pmirGLO‐H19‐Mut and miRNA‐22‐3p measured by a dual‐luciferase reporter assay. ^#^ *P* < .05. (E, F) The levels of H19 and miRNA‐22‐3p in RNA immunoprecipitates are presented as the fold enrichment in Ago2 relative to IgG immunoprecipitates. ^#^ *P* < .05. (G, H) H19 is associated with miRNA‐22‐3p. H19 was pulled down by miRNA‐22‐3p, and the levels of H19 were analysed by RT‐PCR. ^#^ *P* < .05. I, miRNA‐22‐3p is associated with H19. miRNA‐22‐3p could be pulled down by the H19 probe in cardiomyocytes, and the levels of miRNA‐22‐3p were analysed by qRT‐PCR. ^#^ *P* < .05. The values are expressed as the mean ± SD

Ample evidence suggests that lncRNAs in the cytoplasm may exert their biological activities by acting as competing endogenous RNAs to decoy or adsorb miRNAs. To conduct a thorough inquiry on how H19 participates in the regulation of MI, we first used bioinformatics software (RAID V2.0 and Starbase) to screen potential candidate miRNAs that could bind to H19, and miR‐22‐3p was identified. In addition to containing a putative H19 binding site, previous studies have demonstrated that miR‐22‐3p plays a key role in cardiovascular disease, and our data indicated that its level was notably up‐regulated after MI (Figure 3A; *P* < .05). Importantly, as presented in Figure 3A,B, there was a negative correlation between H19 and miR‐22‐3p after MI, namely, enforcing H19 expression significantly decreased miR‐22‐3p levels and vice versa. To further investigate whether H19 could directly interact with miR‐22‐3p, a luciferase construct containing the full‐length H19 sequence or a deletion of the corresponding putative binding sites in H19 was designed (Figure 3C). The results revealed that transfection with the miR‐22‐3p mimic could markedly suppress the luciferase activity of H19, while it had less effect on the mutated form of H19 (Figure 3D; *P* < .05), indicating a specific interaction of H19 and miR‐22‐3p at this putative binding site. It has also been demonstrated that miRNAs exert their function through RNA‐induced silencing complex (RISC), in which the Ago2 protein plays a pivotal role in binding both miRNAs and their corresponding complementary RNA molecules. For further confirmation, a RIP assay was performed to verify whether H19 and miR‐22‐3p were in the same RISC. The results obviously showed preferentially enriched H19 and miR‐22‐3p RNA levels in the Ago2 immunoprecipitates when compared with those in the negative control IgG in cardiomyocytes (Figure 3E,F; ^#^ *P* < .05). Moreover, we also applied biotin‐labelled pull‐down assays to detect whether miR‐22‐3p and H19 could pull down each other in cardiomyocytes. As presented in Figure 3H, the data indicated that H19 was pulled down by wild‐type miR‐22‐3p; however, the mutated type with disrupted binding sites was unable to pull down H19. Similarly, Figure 3I shows that compared with treatment with Bio‐probe NC, treatment with Bio‐H19‐WT significantly increased the enrichment of miR‐22‐3p (*P* < .05), while no significant difference was found in the enrichment of miR‐22‐3p following treatment with Bio‐H19‐Mut (*P* > .05). Collectively, the data above suggest that H19 could directly interact with miR‐22‐3p in rat cardiomyocytes.

### 3.3 miR‐22‐3p knockdown ameliorated myocardial injury caused by MI

**Figure 4:**
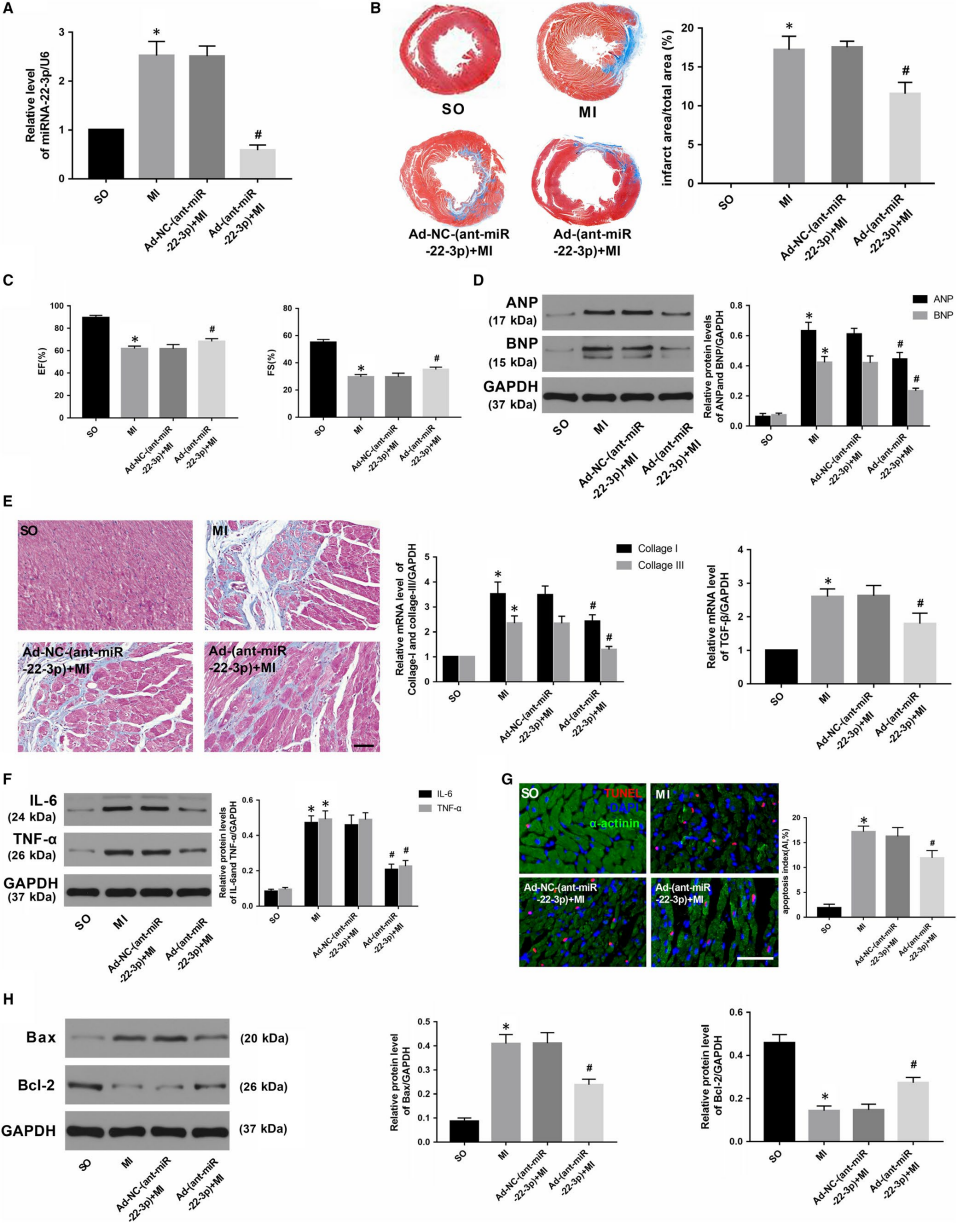
miRNA‐22‐3p knockdown ameliorated AMI‐induced myocardial injury and the consequent maladaptive cardiac remodelling. A, The transfection efficiency of Ad‐(anti‐miR‐22‐3p) was determined by qRT‐PCR. B, Representative Masson's staining images and the infarct rate of the different groups. C, EF and FS 4 wk after AMI in the different groups. D, The expression of ANP and BNP in the different groups. E, Representative images of Masson's staining (scale bar = 50 μm) and the mRNA expression of collagen I, collagen III and TGF‐β. F, The expression of IL‐6 and TNF‐α in the different groups. G, Representative images of TUNEL staining and AI in the different groups (scale bar = 50 μm). H, The expression of Bax and Bcl‐2 in the different groups. The values are expressed as the mean ± SD. *P < .05 versus the SO group; ^#^ *P* < .05 versus the Ad‐NC‐(anti‐miR‐22‐3p)+MI group. The values are expressed as the mean ± SD

To better understand the potential biological function of miR‐22‐3p in the heart, we constructed recombinant adenoviruses containing Antagomir‐22 (Ad‐Antagomir‐22) to knock down the expression of miR‐22‐3p. As shown in Figure 4A, the RNA level of miR‐22‐3p was distinctly down‐regulated after transfection with Ad‐Antagomir‐22 (*P* < .05). Four weeks after MI, the data showed that miR‐22‐3p depletion remarkably minimized myocardial infarct size and improved spoiled cardiac function as well as decreased the extent of cardiac fibrosis when compared with those in the group transfected with negative control adenoviruses (Figure 4B,C,D and 4; *P* < .05). Concomitantly, the inflammatory response was abated, and the cardiomyocyte apoptosis index was reduced in response to miR‐22‐3p knockdown after MI, as evidenced by the changes in inflammation‐related indicators (IL‐6, TNF‐α) and apoptosis markers (Bax, Bcl‐2) (Figure 4F, G, and H; *P* < .05).

### 3.4 KDM3A is a direct target of miR‐22‐3p, and its expression in the heart is regulated by both miR‐22‐3p and lncRNA H19

**Figure 5:**
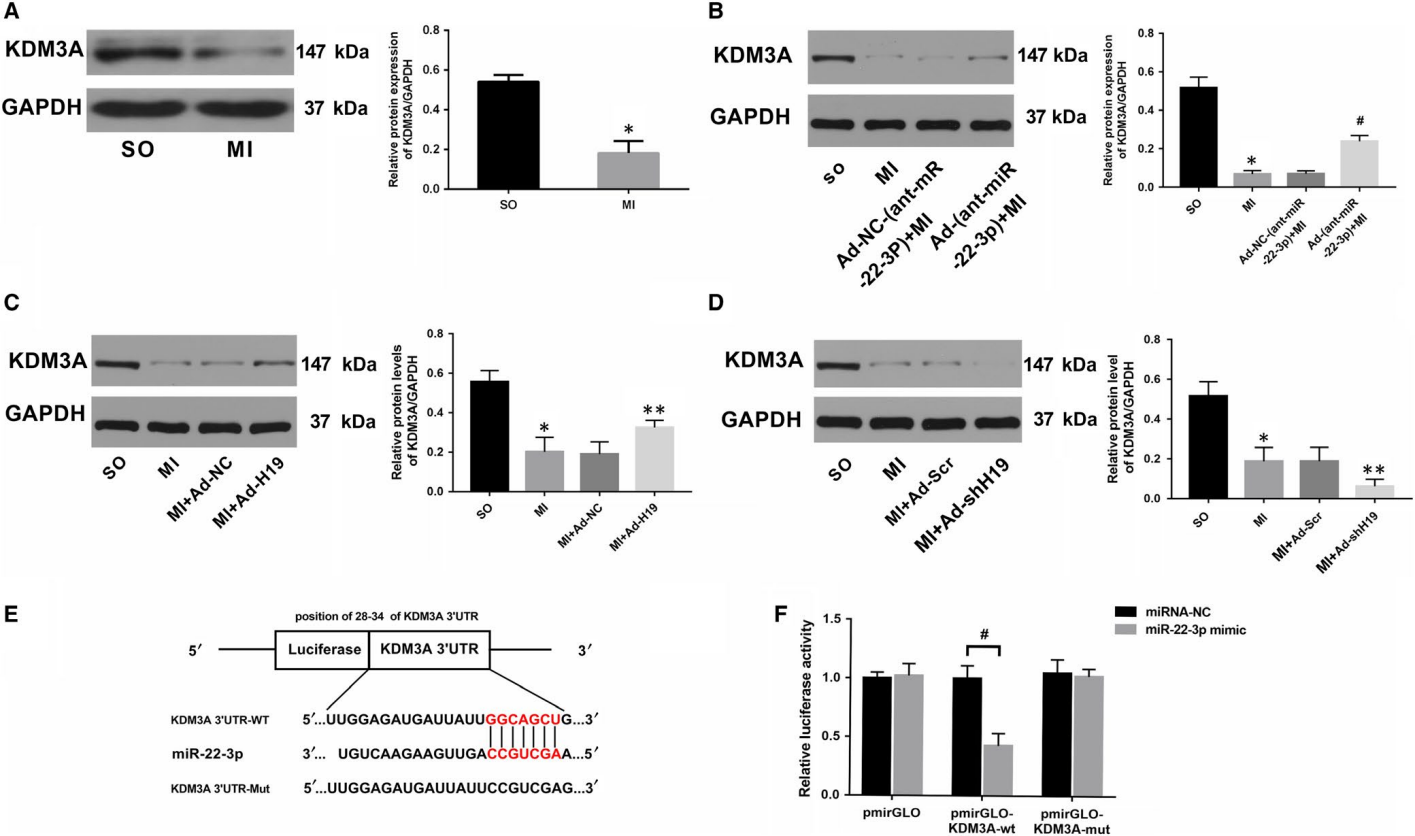
KDM3A is a direct target of miRNA‐22‐3p. A, KDM3A levels in normal myocardial tissue or in the infarction border zone. B, KDM3A levels after down‐regulation of the expression of miRNA‐22‐3p by adenovirus infection. *P < .05 versus the SO group; ^#^ *P* < .05 versus the Ad‐NC‐(anti‐miR‐22‐3p)+MI group. (C, D) Western blot analysis of KDM3A levels after up‐regulation and down‐regulation of H19 expression by adenovirus infection. *P < .05 versus the SO group; ^**^ *P* < .05 versus the control group. E, miRNA‐22‐3p contains a site complementary to KDM3A. F, Luciferase activity of pmirGLO‐KDM3A‐Wt and pmirGLO‐KDM3A‐Mut after miRNA‐22‐3p transfection in HEK293T cells by dual‐luciferase reporter assay. ^#^ *P* < .05. The values are expressed as the mean ± SD

As miRNAs exert their biological effects by binding to the mRNA of their target gene and negatively regulating its expression, determining the downstream protein‐coding gene of miR‐22‐3p is of particular importance. To this end, we once again screened for potential genes that could bind to miR‐22‐3p using bioinformatics software and surprisingly found that the 3ʹ‐UTR of the KDM3A mRNA sequence harboured a putative complementary binding site for miR‐22‐3p. The expression of KDM3A was significantly decreased after MI, while miR‐22‐3p inhibition could obviously up‐regulate its level (Figure 5A,B; *P* < .05). Moreover, the data also indicated that there was a positive relationship between H19 and KDM3A (Figure 5C,D). To confirm whether KDM3A is a bona fide target of miR‐22‐3p, a luciferase reporter assay was performed. HEK293T cells were cotransfected with pmirGLO‐KDM3A‐WT or pmirGLO‐KDM3A‐Mut and miR‐22‐3p mimics or miRNA‐NC. As shown in Figure 5F, miR‐22‐3p mimics dramatically reduced the luciferase activity of the pmirGLO‐KDM3A‐WT group compared with the miRNA‐NC group (*P* < .05). However, neither the miR‐22‐3p mimic nor its negative control evidently influenced luciferase activity in the pmirGLO‐KDM3A‐Mut group (*P* > .05).

### 3.5 KDM3A overexpression alleviated while KDM3A knockout deteriorated myocardial injury induced by MI

**Figure 6:**
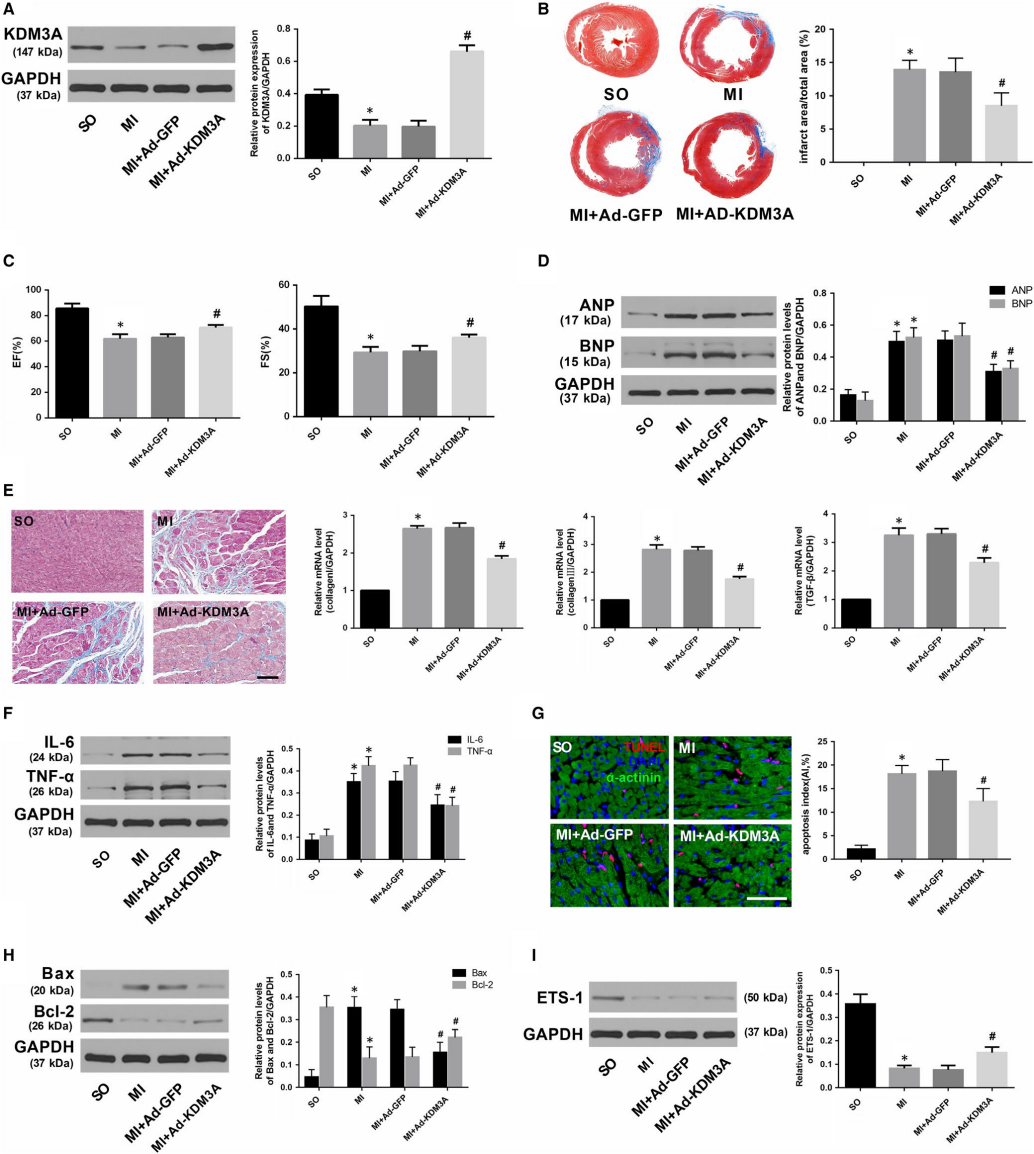
Enforced KDM3A expression obviously mitigated AMI‐induced myocardial injury and subsequent maladaptive cardiac remodelling. A, The transfection efficiency of Ad‐KDM3A was determined by Western blot. B, Representative Masson's staining images of the heart papillary muscle and the infarct rate of the different groups. C, EF and FS of the different groups 4 wk after AMI. D, The expression of ANP and BNP 4 wk after AMI. E, Representative images of Masson's staining (scale bar = 50 μm) and mRNA levels of collagen I, collagen III and TGF‐β. F, The expression of IL‐6 and TNF‐α. G, TUNEL staining and apoptotic index in the different groups (scale bar = 50 μm). (H, I)The expression of Bax, Bcl‐2 and ETS‐1. The values are expressed as the mean ± SD. *P < .05 versus the SO group; ^#^ *P* < .05 versus the MI + Ad‐GFP group

**Figure 7:**
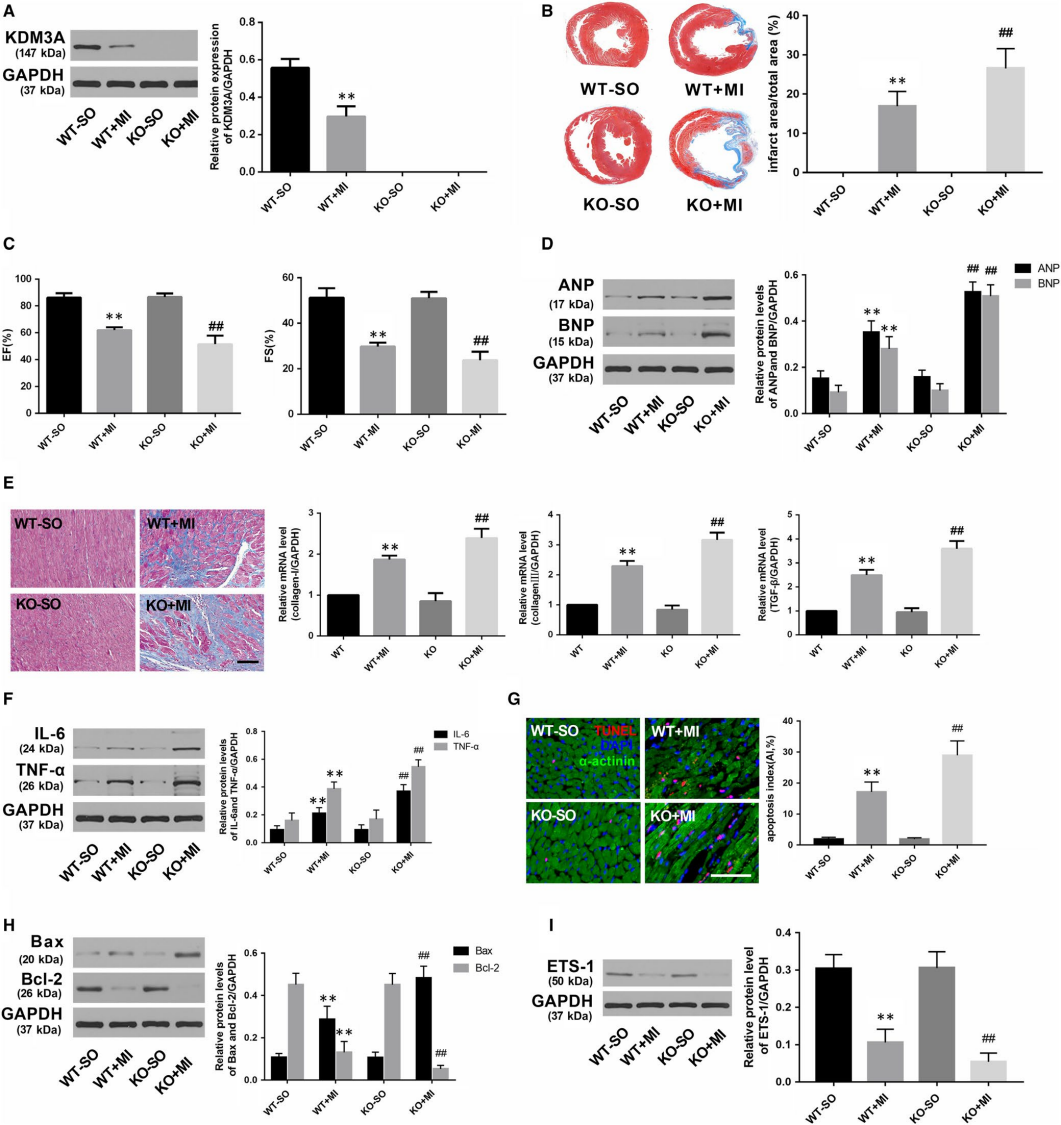
KDM3A knockout obviously aggravated AMI‐induced myocardial injury and the ensuing cardiac remodelling. A, The protein levels of KDM3A in the different groups. B, Representative Masson's staining images of the heart papillary muscle and the infarct rate of the different groups. C, EF and FS in the different groups. D, The levels of ANP and BNP 4 wk after AMI. E, Representative images of Masson's staining (scale bar = 50 μm) and the mRNA expression of collagen I, collagen III and TGF‐β in the different groups. F, The expression of inflammatory cytokines in the different groups. G, Representative images of TUNEL staining and apoptotic index in the different groups (scale bar = 50 μm). (H, I) The expression of apoptosis‐related protein and ETS‐1 in the different groups. The values are expressed as the mean ± SD. **P < .05 versus the WT‐SO group; ^##^ *P* < .05 versus the WT + MI group

To investigate whether KDM3A is involved in MI, another set of controlled gain‐ and loss‐of‐function experiments was performed. Rat hearts were transfected with adenovirus encoding KDM3A (Ad‐KDM3A) to up‐regulate the level of KDM3A (Figure 6A; *P* < .05). KDM3A‐KO rats were used to down‐regulate the expression of KDM3A (Figure 7A; *P* < .05). As presented in Figure 6B,C and 6, KDM3A overexpression significantly reduced infarcted size and improved compromised cardiac function when compared with those in the MI + Ad‐GFP group (*P* < .05). Moreover, enforced KDM3A expression mitigated myocardial fibrosis, decreased the expression of the inflammatory cytokines as well as reduced the number of TUNEL‐positive cells and the levels of pro‐apoptotic proteins (Figure 6E,F,G and H; *P* < .05). In addition, the expression of ETS‐1, which is an important mediator implicated in apoptosis and inflammation and one of the known targets of KDM3A, was also up‐regulated (Figure 6I; *P* < .05). However, the opposite was observed in KDM3A knockout experiments. As shown in Figure 7B,C and 7, compared with the wild‐type rat, knocking out the KDM3A gene augmented the area of myocardial infarction and led to further deterioration of cardiac function post‐MI when compared with those in the WT + MI group (*P* < .05). In addition, KDM3A knockout aggravated myocardial fibrosis, cardiomyocyte apoptosis and the inflammatory response (Figure 7E,F,G and H; *P* < .05). The expression of ETS‐1 was simultaneously down‐regulated (Figure 7I; *P* < .05). The results above suggest the feasibility of KDM3A as a potential mediator in MI.

### 3.6 Enforced expression of miR‐22‐3p abrogated the mitigating effect of lncRNA H19 overexpression on MI and reversed KDM3A levels

**Figure 8:**
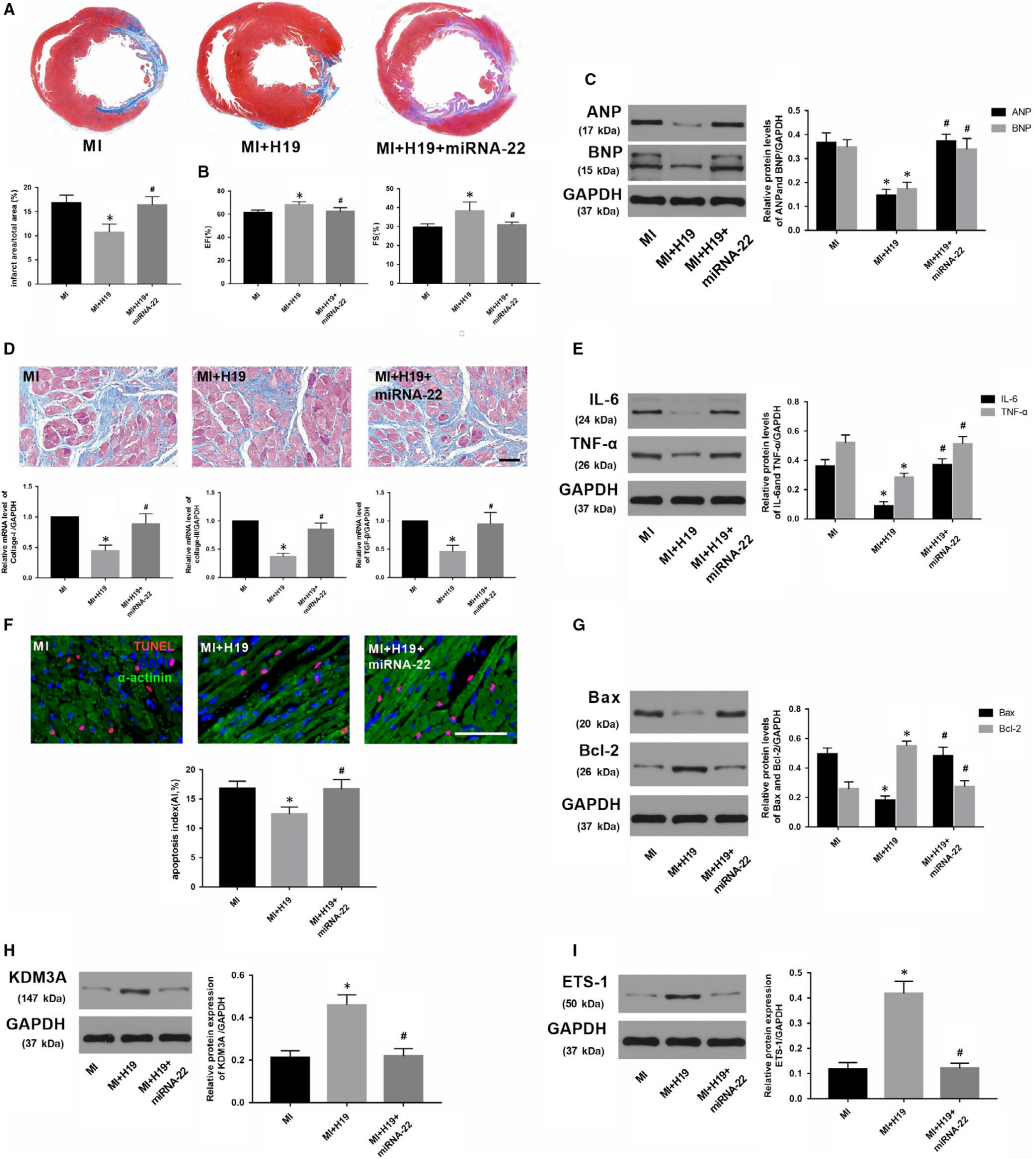
miRNA‐22‐3p overexpression could counteract the beneficial effect of H19 up‐regulation. A, Representative Masson's staining images of the heart papillary muscle cross‐sectional scan and the infarct rate of the different groups. B, EF and FS. C, The expression of ANP and BNP 4 wk after AMI. D, Representative images of Masson's staining (scale bar = 50 μm) and the mRNA expression of collagen I, collagen III and TGF‐β in the different groups. E, The expression of IL‐6 and TNF‐α in different groups. F, Representative images of TUNEL staining and apoptotic index in the different groups. (G, H, I) Western blot analysis of the expression of Bax, Bcl‐2, KDM3A and ETS‐1 in the different groups. The values are expressed as the mean ± SD. *P < .05 versus the MI group; ^#^ *P* < .05 versus the MI + H19 group

As H19 negatively regulates the expression of miR‐22‐3p but positively regulates KDM3A, and an inverse correlation also exists between miR‐22‐3p and KDM3A. Thus, we hypothesized that H19 could regulate KDM3A expression by sponging miR‐22‐3p in MI. A rescue experiment was performed to corroborate this hypothesis. First, a recombinant adenovirus (Ad‐miR‐22) was constructed for miR‐22‐3p overexpression. Thereafter, the transfection combinations were conducted five days before MI surgery, and Ad‐H19 and/or Ad‐miR‐22 were transfected to up‐regulate the expression of H19 and/or miR‐22‐3p, respectively. As expected, compared with the MI rat that was transfected with Ad‐H19, enforced expression of miR‐22‐3p significantly counteracted the effect of H19 on reducing myocardial infarction size (Figure 8A; *P* < .05). In addition, miR‐22‐3p overexpression also partly offset the cardiac function improvement and myocardial fibrosis reduction induced by H19 (Figure 8B,C and 8; *P* < .05). Similarly, compared with those in the group transfected only with Ad‐H19, enforced expression of miR‐22‐3p obviously facilitated the expression of the pro‐inflammatory mediators as well as increased the number of TUNEL‐positive cells and the levels of apoptosis‐associated proteins (Figure 8E,F and G; *P* < .05). Furthermore, miR‐22‐3p up‐regulation also counteracted the induction of KDM3A and ETS‐1 by H19 overexpression (Figure 8H and 8; *P* < .05). Collectively, the data above demonstrated that H19 regulates the expression of KDM3A to alleviate MI‐induced myocardial injury and cardiac remoulding in a miR‐22‐3p‐dependent manner.

## 4 DISCUSSION

Although extensively applied reperfusion therapy has greatly improved patient outcome, AMI is still correlated with high mortality, and the overall clinical prognosis remains unsatisfactory.^21^ In addition to acute myocardial ischaemic damage and reperfusion injury, heart failure triggered by the ensuing maladaptive ventricular remodelling can be a truly difficult issue to tackle.^22^ Further understanding the molecular and cellular mechanisms of AMI and discovering new impactful therapeutic targets to prevent this acute cardiovascular event and the concomitant complications are essential for cardiovascular biology research. In the present study, we established an in vivo rat AMI model to investigate the functional role of lncRNA H19 in the pathological process of AMI. Our data showed that H19 was significantly down‐regulated after MI. Enforced expression of H19 could remarkably reduce myocardial infarction size, improve long‐term cardiac function and relieve cardiac fibrosis by mitigating myocardial apoptosis and decreasing inflammation, while H19 knockout exacerbated the symptoms. In addition, we also explored the potential underlying molecular mechanism through which H19 is involved in the pathological progression of AMI. Our results clearly demonstrated that H19 could function as an endogenous sponge to competitively bind to miR‐22‐3p and up‐regulate the expression of KDM3A, which consequently protected myocardium from AMI‐induced injury.

Although only a very small percentage of lncRNAs have been identified and investigated experimentally so far, it has been well confirmed that lncRNAs are implicated in multiple complex biological processes.^6^, ^23^, ^24^ LncRNAs are so versatile that they have been proposed to possess the capability to interact with protein, DNA and various kinds of RNAs to elicit gene activation or inhibition effects.^25^ Recently, accumulating evidence has been presented in the literature, providing verification of the pivotal roles played by lncRNAs in the homeostasis of the cardiovascular system. Aberrant expression of LncRNAs such as Fendrr, Bvhta and KCNQ1OT1 may lead to congenital cardiovascular diseases and malformations.^10^ Besides, ANRIL, MIAT and MALAT1 have been demonstrated to play crucial roles in ischaemic heart disease.^26^ In addition, enforced expression of Mhrt ameliorated cardiac hypertrophy,^27^ while CHRF up‐regulation exacerbated cardiomyocyte hypertrophy.^28^ Moreover, lncRNAs might serve as new biomarkers of heart diseases. Mitochondrial lncRNA LIPCAR was identified as a biomarker of ventricular remodelling after MI and a predictor of death in patients with heart failure, which confirms the principle that plasma lncRNAs might be used as biomarkers of prognosis in cardiovascular diseases.^29^

LncRNA H19 was the first imprinted lncRNA identified. In contrast to many other lncRNAs that are prone to variation, H19 possessing a highly evolutionarily conserved secondary structure and a sequence consistent among multiple species with only a few mutations in exon regions.^9^, ^12^ As the vast disparities of its expression level in the cardiovascular system before and after birth as well as under normal and pathological conditions, H19 has recently drawn considerable interest among researchers investigating cardiovascular disease.^12^, ^14^ In the pathological process of myocardial ischaemia‐reperfusion injury, Wang et al indicated that H19 could antagonize cardiomyocyte necrosis by directly binding to miR‐103/107 and regulating FADD expression.^11^ Likewise, Zhang et al also verified that H19 could protect senescent cardiomyocytes against hypoxia‐reoxygenation injury by competitively binding to miR‐29b‐3p.^30^ Besides, Hadji demonstrated that dysregulation of DNA methylation in the H19 promoter was associated with the abnormal mineralization of the aortic valve by silencing the NOTCH1 pathway.^13^ In addition to being an independent lncRNA, H19 also carries a miRNA‐containing hairpin, which could serve as a template to encode miR‐675 and inhibit the expression of CaMKIIδ, thereby exerting an anti‐cardiac hypertrophy effect.^12^ However, the potential role of H19 in AMI is still far from clear. Inflammation and apoptosis are two crucial factors that contribute to AMI and the subsequent cardiac remoulding.^1^, ^5^, ^19^ Interestingly, investigators have verified that H19 is a key mediator involved in myocardial inflammatory reactions and apoptosis.^12^, ^30^, ^31^ Consistent with these previous studies, the data from our study clearly illustrated that H19 up‐regulation not only significantly reduced the expression of the pro‐inflammatory factors IL‐6 and TNF‐α but also decreased the number of TUNEL‐positive cells and the ratio of Bax/Bcl‐2 in the context of AMI. Therefore, enforced H19 expression resulted in smaller infract size, better cardiac performance and less myocardial fibrosis when compared with those in the control group. Nevertheless, H19 knockdown exhibited a completely opposite trend. Therefore, based on the aforementioned exploration of the literature and our present study, it is reasonable to infer that H19 might represent a new molecule for AMI targeted therapy.

If we are only at the beginning of understanding the role of H19 in AMI, we are even further away from comprehending the underlying mechanism. The competing endogenous RNA (ceRNA) theory has shed new light on how H19 participates in the pathological process of AMI.^32^ There is wide consensus that lncRNAs may exert their biological effects by acting as endogenous decoys to sponge miRNAs, which consequently segregates miRNAs from their target mRNAs.^20^, ^25^ In recent decades, the vital function of miRNAs in cardiovascular disease has been well characterized. Among them is miRNA‐22‐3p, which was shown to be enriched in strained muscle tissues and has been confirmed to be involved in various cardiovascular disorders through regulating inflammation, apoptosis, oxidative stress and autophagy.^33^, ^34^, ^35^, ^36^ In our present study, we transfected cardiomyocytes with adenovirus containing siRNA to down‐regulate the miRNA‐22‐3p level to explore its effect on AMI. As expected, miRNA‐22‐3p knockdown significantly decreased the MI‐post infarction area and improved cardiac function as well as alleviated myocardial fibrosis. Moreover, the inflammatory response and apoptosis were also mitigated. Therefore, miRNA‐22‐3p inhibition could ameliorate myocardial injury. An interesting phenomenon is that in contrast to the expression status of H19 in the heart, the level of cardiac miR‐22‐3p is much higher in mature cardiomyocytes when compared with those of embryonic and neonatal hearts.^33^ Our data revealed a negative correlation between H19 and miRNA‐22‐3p levels in AMI, and bioinformatics analysis also showed that H19 contains a putative binding site for miRNA‐22‐3p. In addition, previous studies performed by Wang and colleagues have indicated that H19 could modulate H2O2‐induced nucleus pulposus cell dysfunction by regulating miRNA‐22‐3p.^37^ Consistent with their findings, the results from the luciferase reporter assay clearly suggested that miRNA‐22‐3p was a direct target of H19. Furthermore, RIP and RNA pull‐down assays reconfirmed the physical interaction between H19 and miRNA‐22‐3p in cardiomyocytes. After considering these results, we speculate that H19 might directly bind to miRNA‐22‐3p and elicit cardioprotective effects by suppressing the biological activity of miRNA‐22‐3p in the context of AMI.

It has been verified that miRNAs mainly exert their regulatory effects at the transcriptional level by either promoting mRNA degradation or suppressing mRNA transcriptional activity, thereby inhibiting the expression of target proteins.^20^, ^25^, ^32^, ^38^ As a result, we placed particular emphasis on identifying the target protein of miRNA‐22‐3p in AMI. In the past several years, our research group has endeavoured into the study of the histone demethylase KDM3A, and we firmly believe it is a key node in the cardiovascular regulatory network. Our initial studies showed that KDM3A could promote vascular neointimal hyperplasia in diabetic rats via the AGTR1 and ROCK2 signalling pathways.^15^ KDM3A inhibition could also attenuate high concentration insulin–induced vascular smooth muscle cell injury by suppressing MAPK/NF–κB pathways.^17^ In addition, our latest findings suggest that KDM3A is involved in diabetic cardiomyopathy and is a crucial factor in mediating myocardial ‘Metabolic Memory’ injury in diabetes by facilitating NF‐κB/p65 transcriptional activities. Moreover, our unpublished research also indicated that KDM3A played an important role in myocardial ischaemia‐reperfusion injury. More recently, other researchers, such as Zhang et al recently provided solid genetic and biochemical evidence that pharmacological targeting of KDM3A is an effective strategy to counteract left ventricular hypertrophy.^18^ However, its potential role in AMI has not been explicitly delineated. In our present study, the gain‐ and loss‐of‐function experiment showed that KDM3A overexpression significantly alleviated inflammatory responses, reduced apoptosis, minimized infarct size and improved cardiac function. Meanwhile, ETS‐1, which is a pivotal regulator involved in inflammation, ROS and apoptosis were also up‐regulated.^39^, ^40^ However, the opposite was true in KDM3A‐knockout rats. We and others have found that ETS‐1 is a downstream target of KDM3A,^41^ and our latest experiment verified that KDM3A could directly bind to the promoter of ETS‐1 and control H3K9 methylation in myocardial ischaemia‐reperfusion injury. KDM3A overexpression displayed higher ETS‐1 transcriptional activity concomitant with reduced inflammation, ROS and apoptosis. As both inflammation and apoptosis are critical underlying mechanisms of AMI and cardiac remoulding, we speculate that KDM3A might be involved in the pathological process of AMI by regulating the expression of ETS‐1. A previous study published by Parrish and colleagues uncovered that KDM3A is a miRNA‐22‐3p‐regulated tumour promoter in Ewing sarcoma.^42^ Here, our data not only demonstrated a negative correlation between miRNA‐22‐3p and KDM3A in MI, but the subsequent luciferase reporter assay also confirmed that miRNA‐22‐3p could directly combine with KDM3A mRNA. Therefore, the data above indicated that miRNA‐22‐3p might participate in AMI through regulating the expression of KDM3A. Intriguingly, we found that H19 shares the same miR‐22‐3p binding sites with the KDM3A mRNA 3’‐UTR. To verify that miRNA‐22‐3p is a specific mediator between H19 and KDM3A, we then performed a rescue experiment to up‐regulate the expression of H19 and miRNA‐22‐3p in myocardium at the same time. The result from this study indicated that enforced miRNA‐22‐3p expression could partly counteract the beneficial cardiac protective effect of H19 overexpression and reverse the protein levels of KDM3A and ETS‐1. Based on the above results, we concluded that H19 could sponge miRNA‐22‐3p to ameliorate AMI‐induced myocardial damage by up‐regulating KDM3A.

## 5 CONCLUSION

In summary, we identified that lncRNA H19 expression was significantly down‐regulated after AMI. Functional experiments showed that H19 could reduce myocardial apoptosis and decrease inflammation, thereby minimizing infarct size and improving cardiac function as well as inhibiting maladaptive myocardial remodelling. Mechanistically, we demonstrated that H19 plays a ceRNA role in regulating KDM3A expression by competitively binding to miRNA‐22‐3p. In general, our present study provided new insight into the post‐transcriptional regulatory mechanism of H19 implicated in AMI and established the potential value of the H19/miRNA‐22‐3p/KDM3A pathway in preventative treatment for AMI. However, it is noteworthy that one lncRNA may interact with multiple miRNAs and one miRNA may act on several target genes. miRNA‐22‐3p possess several potential targets in ischaemic myocardial disease (Table 1). In addition, it has also been reported that some transcription factors such as TGF‐β and DNA methyltransferase such as DNMT‐1 might serve as the upstream regulator of H19 and participate in the pathological process of other diseases. As a result, further studies are still needed to establish if there are other direct links between H19 and other signalling pathways that participate in the pathological process of AMI.

**Table 1:** The potential targets of miR‐22‐3p that may involve in myocardial infarction

Pathological model	The expression of miR‐22‐3p	Target gene of miR‐22‐3p	Underlying mechanism
Hypoxic‐treated cardiomyocytes H9c2	Up‐regulated	PI3K and JAK1	miR‐22‐3p overexpression facilitates apoptosis
Mice myocardial infarction model	Up‐regulated	Cav3	miR‐22‐3p overexpression facilitates cardiac fibrosis
Rat myocardial infarction model	Up‐regulated	KDM3A	miR‐22‐3p overexpression facilitates apoptosis and inflammatory reaction
Rat myocardial ischaemia‐reperfusion model and H9c2 cardiomyocytes hypoxia/reoxygenation model	Up‐regulated	Sirt1 and PGC1α	miR‐22‐3p overexpression facilitates mitochondrial oxidative
Patients with coronary artery disease	Up‐regulated	MCP‐1	miR‐22‐3p overexpression facilitates inflammatory reaction
Starvation‐induced neonatal rat cardiomyocytes injury	Down‐regulated	p38α	miR‐22‐3p overexpression promotes autophagy and inhibits apoptosis

## CONFLICT OF INTEREST

The authors declare that they have no competing interests.

## AUTHOR CONTRIBUTIONS

Bofang Zhang, Jing Chen and Hong Jiang have made substantial contributions to conception and design; Bofang Zhang and Jing Chen have done acquisition of data; Qi Hu, Shuo Yang and Gen Liu have done the analysis and interpretation of the data; Bofang Zhang and XiaoPei Liu were involved in drafting the manuscript; Hong Jiang and Jing Chen revised it critically for important intellectual content.

## Data Availability

The data sets generated during and/or analysed during the current study are available from the corresponding author on reasonable request.
