# LncRNA H19 ameliorates myocardial infarction‐induced myocardial injury and maladaptive cardiac remodelling by regulating KDM3A

**DOI:** 10.1111/jcmm.17753

**Published:** 2023-06-16

In Bofang Zhang et al.,^1^ the published article contains errors in Figures 6 and 8. The correct images are shown below. The authors confirm all results and conclusions of this article remain unchanged.

**FIGURE 6:**
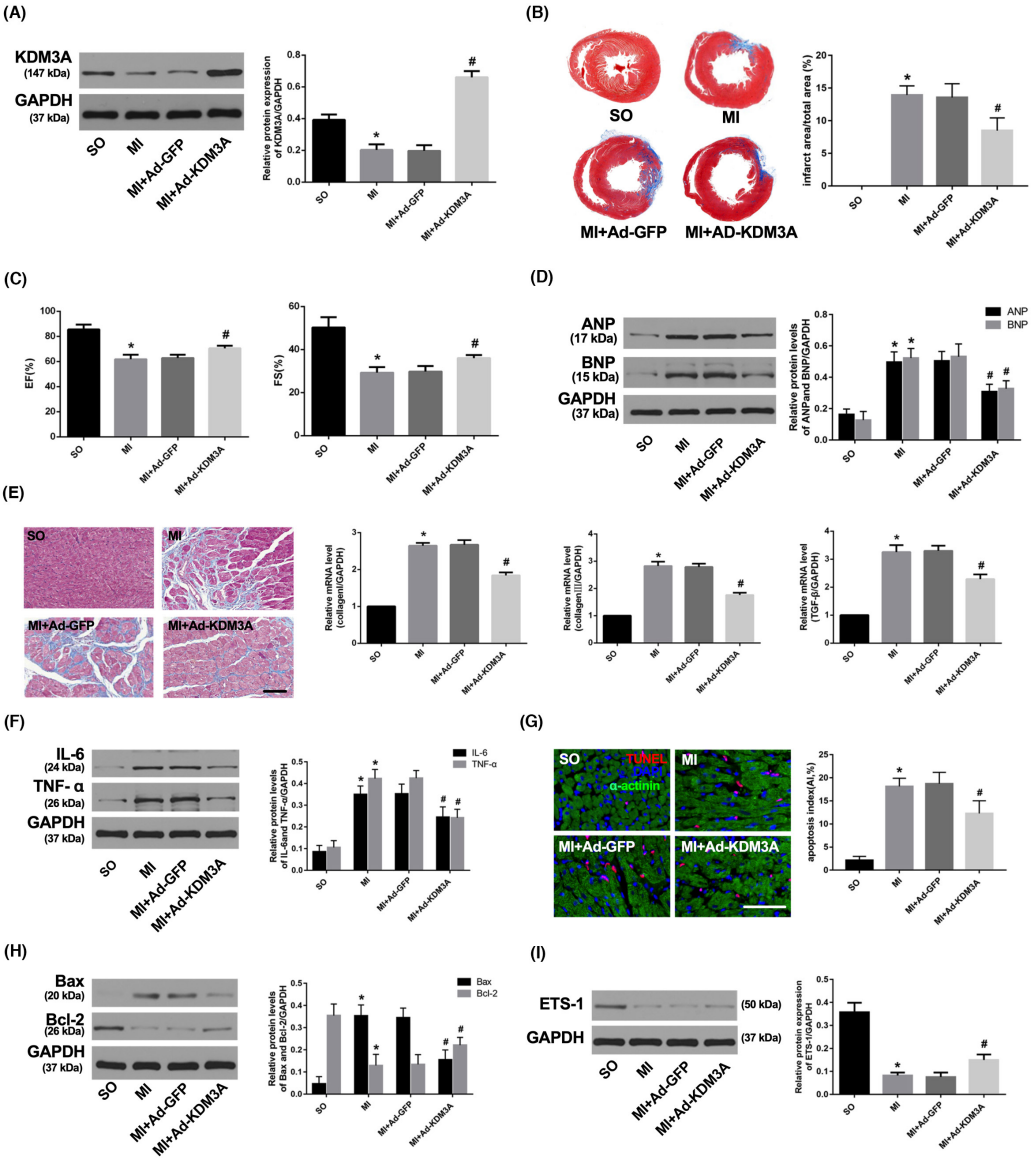
Enforced KDM3A expression obviously mitigated AMI‐induced myocardial injury and subsequent maladaptive cardiac remodelling. (A) The transfection efficiency of Ad‐KDM3A was determined by Western blot. (B) Representative Masson's staining images of the heart papillary muscle and the infarct rate of the different groups. (C) EF and FS of the different groups 4 week after AMI. (D) The expression of ANP and BNP 4 week after AMI. (E) Representative images of Masson's staining (scale bar = 50 μm) and mRNA levels of collagen I, collagen III and TGF‐β. (F) The expression of IL‐6 and TNF‐α. (G) TUNEL staining and apoptotic index in the different groups (scale bar = 50 μm). (H, I) The expression of Bax, Bcl‐2 and ETS‐1. The values are expressed as the mean ± SD. *p < 0.05 versus the SO group; ^**#**^ *p* < 0.05 versus the MI + Ad‐GFP group.

**FIGURE 8:**
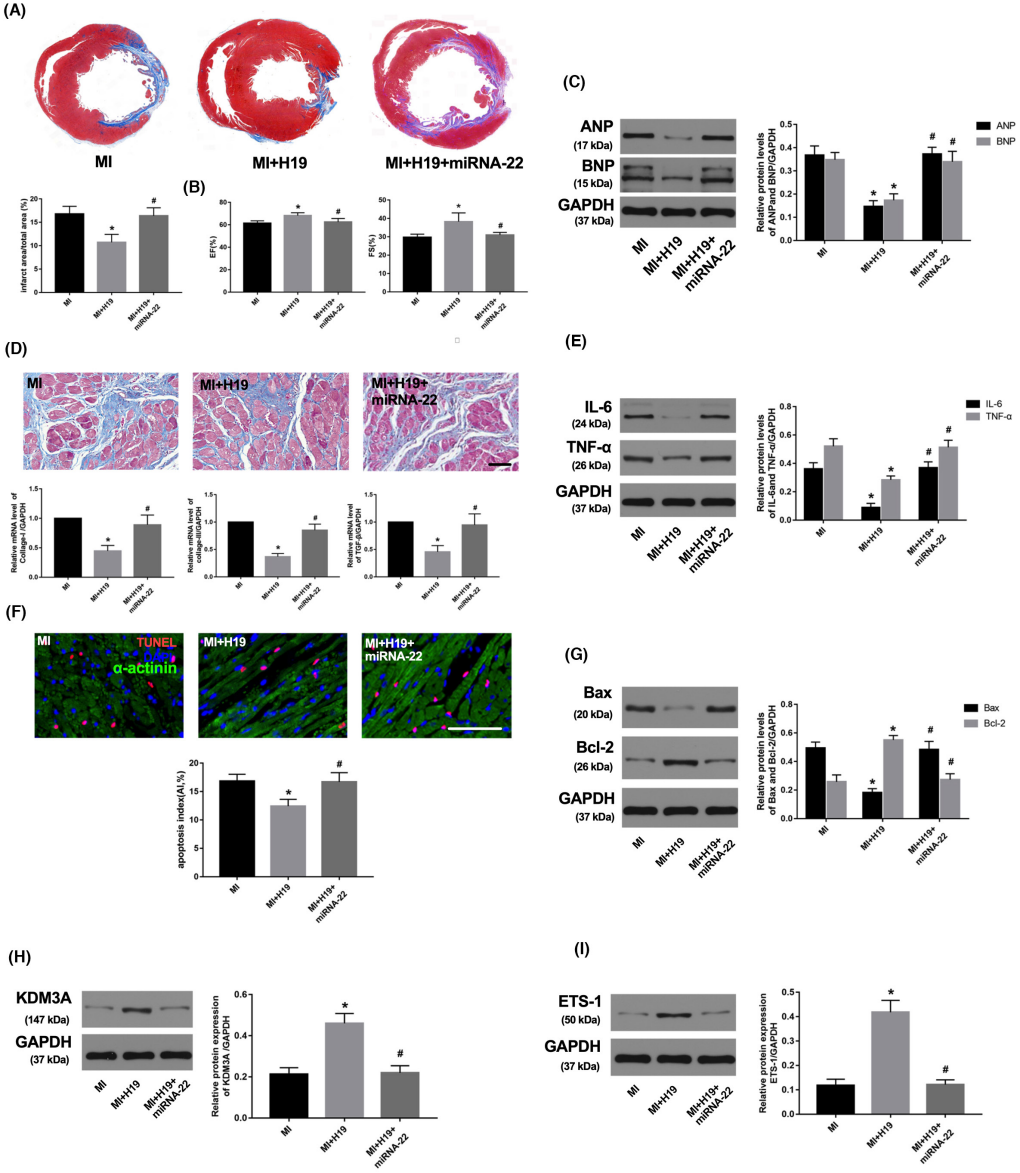
miRNA‐22‐3p overexpression could counteract the beneficial effect of H19 up‐regulation. (A) Representative Masson's staining images of the heart papillary muscle cross‐sectional scan and the infarct rate of the different groups. (B) EF and FS. (C) The expression of ANP and BNP 4 week after AMI. (D) Representative images of Masson's staining (scale bar = 50 μm) and the mRNA expression of collagen I, collagen III and TGF‐β in the different groups. (E) The expression of IL‐6 and TNF‐α in different groups. (F) Representative images of TUNEL staining and apoptotic index in the different groups. (G– I) Western blot analysis of the expression of Bax, Bcl‐2, KDM3A and ETS‐1 in the different groups. The values are expressed as the mean ± SD. *p < 0.05 versus the MI group; ^**#**^ *p* < 0.05 versus the MI + H19 group.
